# Supplementary material for: Trends in U.S. self-reported health and self-care behaviors during the COVID-19 pandemic
Source: PLoS One. 2023 Sep 19;18(9):e0291667. doi: 10.1371/journal.pone.0291667 (PMC10508610; doi:10.1371/journal.pone.0291667)
Supplement: S1 File — (DOCX) [file pone.0291667.s001.docx]

**Appendix A1: National trends in exercise participation, 2016-2020**

**
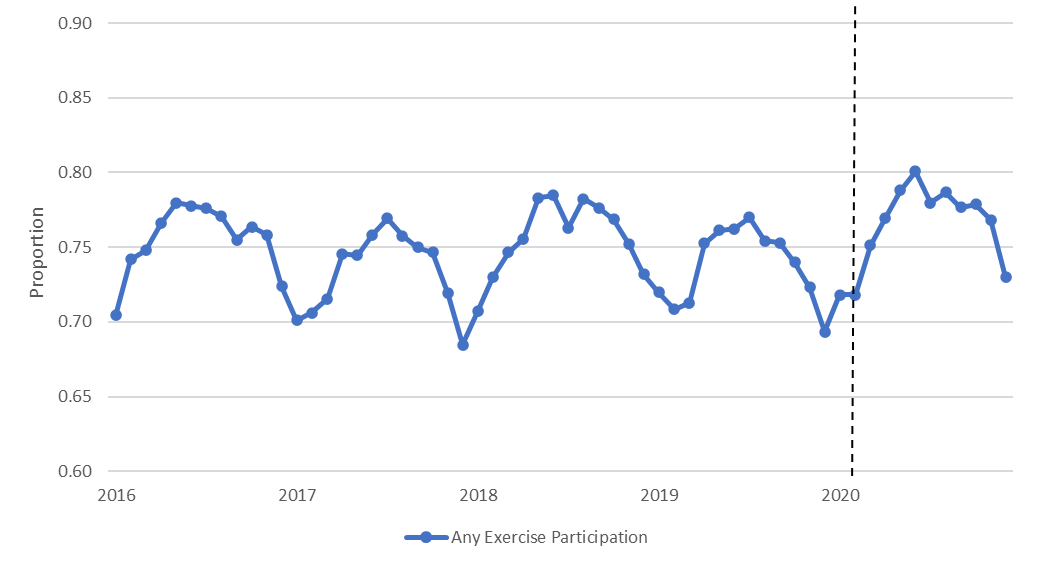
**

**Source:** Authors' analysis of data from the 2016-2020 BRFSS. **Notes:** The figure displays monthly unadjusted trends in the proportion of respondents reporting participation in any exercise, accounting for BRFSS post-stratification weights. The vertical dashed line indicates February 2023.

**Appendix A2: National trends in sleep hours per day, 2016-2020**

**
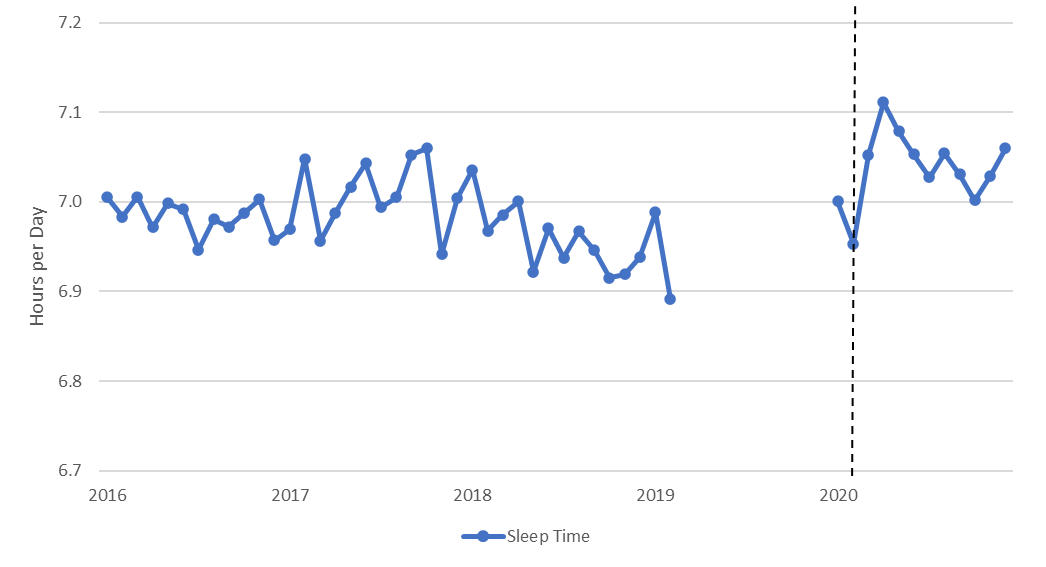
**

**Source:** Authors' analysis of data from the 2016-2020 BRFSS. **Notes:** The figure displays monthly unadjusted trends in reported sleep hours per day, accounting for BRFSS post-stratification weights. The vertical dashed line indicates February 2023. This question was not asked in 2019.

**Appendix A3: National trends in alcohol consumption, 2016-2020**

**
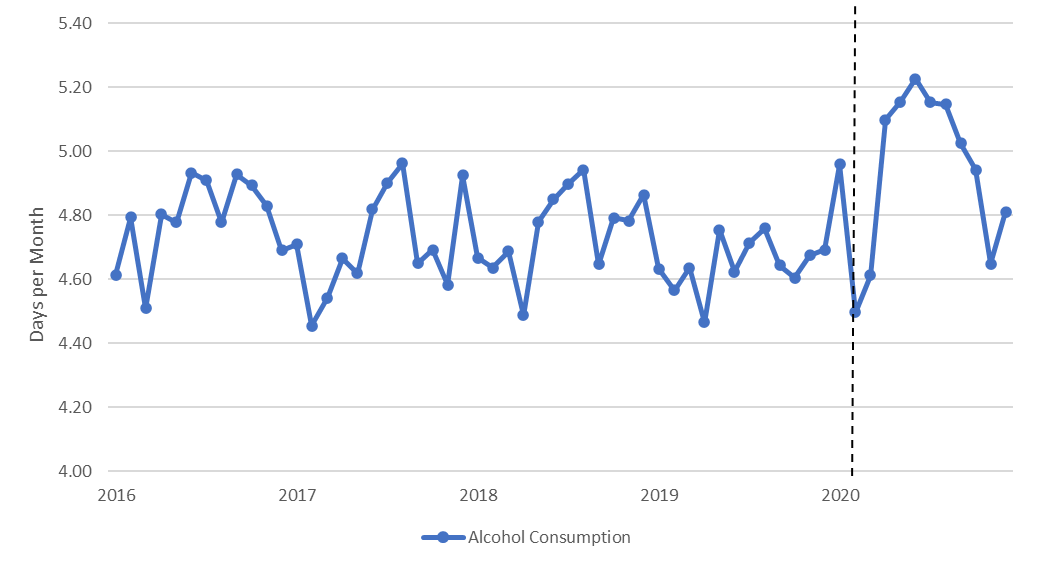
**

**Source:** Authors' analysis of data from the 2016-2020 BRFSS. **Notes:** The figure displays monthly unadjusted trends in the number of days in the past month when alcohol was consumed, accounting for BRFSS post-stratification weights. The vertical dashed line indicates February 2023.

**Appendix A4: National trends in tobacco smoking status, 2016-2020**

**
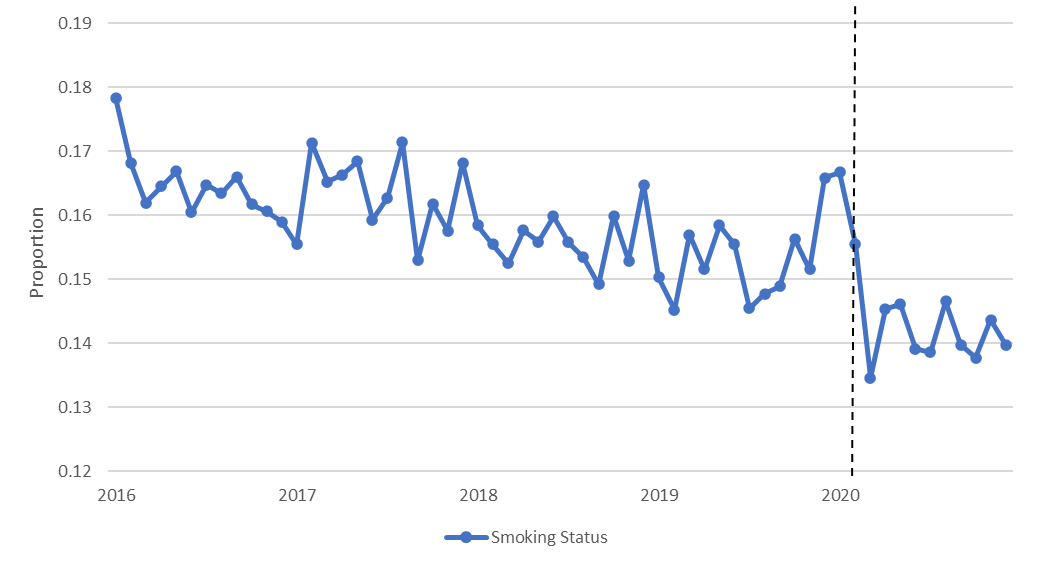
**

**Source:** Authors' analysis of data from the 2016-2020 BRFSS. **Notes:** The figure displays monthly unadjusted trends in the proportion of respondents who report smoking every day or some days, accounting for BRFSS post-stratification weights. The vertical dashed line indicates February 2023.
