# Supplementary material for: Trends in U.S. self-reported health and self-care behaviors during the COVID-19 pandemic
Source: PLoS One. 2023 Sep 19;18(9):e0291667. doi: 10.1371/journal.pone.0291667 (PMC10508610; doi:10.1371/journal.pone.0291667)
Supplement: Attachment — Submitted filename: PLOS Response Letter 2023 08 06.pdf [file pone.0291667.s003.pdf]

VANDERBILT UNIVERSITY School of Medicine

Department of Health Policy

August 6th, 2023

Dear Dr. Roblin,

Thank you for the opportunity to revise and resubmit our manuscript, "Trends in U.S. self-reported health and self-care behaviors during the COVID-19 pandemic" for consideration at *PLOS ONE*. The reviewer's and editor's comments have been very helpful and strengthened the paper. Our responses are below.

Sincerely,

Kevin N. Griffith, Ph.D.
Department of Health Policy
Vanderbilt University School of Medicine

Editorial comments:

1) *Please ensure that your manuscript meets PLOS ONE's style requirements, including those for file naming.*

Done.

2) *Thank you for stating in your Funding Statement: "KNG's effort was supported in part by a grant from the U.S. Agency for Healthcare Research & Quality (https://www.ahrq.gov/, K12 HS026395)." Please provide an amended statement that declares *all* the funding or sources of support (whether external or internal to your organization) received during this study, as detailed online in our guide for authors at http://journals.plos.org/plosone/s/submit-now. Please also include the statement "There was no additional external funding received for this study." in your updated Funding Statement.*

Thank you, an amended statement would be: "KNG's effort was supported in part by a grant from the U.S. Agency for Healthcare Research & Quality (https://www.ahrq.gov/, K12 HS026395). There was no additional external funding received for this study." Apologies, but I did not see a place to included this in the submission portal. There is a "Current Funding Sources List" where I added the AHRQ grant details, but there is no free-text section – please advise!

3) *We note that you have stated that you will provide repository information for your data at acceptance. Should your manuscript be accepted for publication, we will hold it until you provide the relevant accession numbers or DOIs necessary to access your data. If you wish to make changes to your Data Availability statement, please describe these changes in your cover letter and we will update your Data Availability statement to reflect the information you provide.*

VANDERBILT UNIVERSITY

2525 West End Avenue
Suite 1275
Nashville, TN 37203

www.vumc.org/health-policy/
tel 615.322.1324

We have modified the Data Availability statement to now read:

"Our analytic dataset and R scripts are available within an open access Mendeley Data Repository, accessible at https://data.mendeley.com/datasets/nfvpcd9wpf."

This statement has also been added to the end of our Methods section in the manuscript.

4) *Please amend the manuscript submission data (via Edit Submission) to include author Morgan Reinhart.*

Done.

5) *Clearly indicate in the limitation subsection that measurements of outcomes are only based on days without considering "extents: e.g., perceived severity and frequency were one of the missed good measurement indicators for health outcomes like sleep, exercise, etc.*

This is an excellent point, and our revised Study Limitations includes the following passage:

"Third, our outcomes do not capture the severity of health challenges (e.g., minor versus major depression) or the quality of health behaviors (e.g., exercise duration)."

6) *Clearly indicate in the limitation subsection that self-reported health outcomes overly represented by sleep and exercise while omitting access to counseling and communication services, nutrition, engagement in discussions, etc. during Covid-19*

Thank you, we have added the following passage to our revised Study Limitations:

"Lastly, the BRFSS core survey does not contain measures of social engagement, nutrition or access to behavioral health services that may have important implications for physical and mental health."

Additionally, we have added two additional behavioral outcomes to our analysis: alcohol consumption and smoking status.

7) *Clearly indicated in the limitations subsection that measurements of self-reports should have better done by scales*

Thank you, we believe we have addressed this in our response to #5.

8) *Why high income is associated to poor health outcome? Check them against two things: 1) whether measurement gaps exist, 2) if there has been extra expectation experienced by those who have higher income that could link to poor health outcomes.*

Thank you for this question. Table 2 demonstrates that income is *negatively* associated with both poor physical and mental health days at base. Table 3 demonstrates that compared to lower income groups, higher income groups experienced larger increases in mental health days and smaller increases in poor physical health days. We have added the following to our revised Discussion section (citations in text):

"A discussion of the shared and unique challenges faced by each population group during the pandemic is outside the scope of this work. However, prior studies have found that employed individuals experienced heightened fears of COVID-19 exposure and contagion of themselves and their families, inability to secure childcare due to school closures, job insecurity, or difficulty transitioning to remote work during shelter-in-place orders."

<u>Reviewer #1 comments:</u>

9) *In the discussion, I think the authors could discuss their findings a bit more and perhaps the implications for mental health and physical health of the US population. Findings might also be linked to public health initiatives or policy. In addition, were any findings surprising or unexpected (such as increase in physical health among all respondents, which seems to be the only consistent demography response).*

Thank you for this suggestion, our revised Discussion now reads:

"These results may seem counterintuitive; however, our findings comport with prior work documenting positive changes in health and health behaviors during economic downturns. Unemployment spells and economic downturns are associated with temporary reductions in smoking, alcohol consumption, obesity, physical inactivity, and air pollution. We found that sleep and exercise behaviors improved for every subgroup; increases in time spent at home due to COVID-19 mitigation efforts (e.g., shelter in place orders) may have allowed individuals to devote more time to self-care activities. However, these benefits may lack durability in the face of extended lockdowns and associated social isolation. Future research should examine whether our findings replicate in international contexts or represent idiosyncrasies of U.S. workplace culture."

10) *Also in the discussion, the authors might discuss how the increases in sleep and physical activity might be related to the increase in time available for such activities for those populations. What does that say about workplace culture and life in the US? While the authors are correct in restricting their discussion to associations only, I think some speculation or perhaps a further research section might allow them to engage with the "why did we find this?" portion of this paper that is somewhat missing.*

Thank you, we agree and have added the following passage to our revised Discussion:

"However, these benefits may lack durability in the face of extended lockdowns and associated social isolation. Future research should examine whether our findings replicate in international contexts or represent idiosyncrasies of U.S. workplace culture."

11) *Figure 1 is very good and shows a distinct trend in poor mental health days increasing dramatically in 2020, but the increase is linked to an initial decrease in early 2020. Further, looking backward, from 2016 to 2019, there was a steady rise in poor mental health days. If the pandemic had not happened,*

would poor mental health days still have increased (it seems like, visually at least, that the increase was happening prior to the pandemic). It might be worth considering a comparison of the slopes for the change over time before and after the pandemic. Perhaps COVID-19 merely brought attention to an issue that has been building in the US behind the scenes.

Thank you for this discussion! While our data were monthly, the graph was generated using quarterly estimates to provide smoother trend lines. However, this led to the initial pandemic month (March 2020) being included in the first quarter of 2020. This erroneously made it appear that some of the declines (especially for poor mental health days) started to occur prior to the pandemic. We have regenerated Figure 1 to use monthly data, and added a vertical dashed line indicating February 2020.

Our revised Discussion also addresses this important point (citations in main text):

"Furthermore, prior studies often assessed mental health of population subgroups at a single point in time, without controlling for pre-existing trends. For instance, our results comport with prior research using Healthy Minds study data from 2013 to 2021; the authors concluded that the increased prevalence of mental health problems among college students during COVID-19 represented a continuation of pre-existing time trends rather than a unique spike. Indeed, our results in Figure 1 suggests the decline in poor mental health days was a temporary deviation from an increasing national trend."

12) *To me, Figure one shows the strongest association for changes in physical health, which is a very interesting finding since many people speculate that physical health deteriorated for many during the pandemic. Your study would suggest otherwise, and the discussion should state this and suggest why.*

Thank you, we have addressed this in our response to #9. Our revised Discussion now reads (citations in manuscript):

"These results may seem counterintuitive; however, our findings comport with prior work documenting positive changes in health and health behaviors during economic downturns. Unemployment spells and economic downturns are associated with temporary reductions in smoking, alcohol consumption, obesity, physical inactivity, and air pollution. We found that sleep and exercise behaviors improved for every subgroup; increases in time spent at home due to COVID-19 mitigation efforts (e.g., shelter in place orders) may have allowed individuals to devote more time to self-care activities. However, these benefits may lack durability in the face of extended lockdowns and associated social isolation. Future research should examine whether our findings replicate in international contexts or represent idiosyncrasies of U.S. workplace culture."

<u>Reviewer #2 comments:</u>

13) The term 'self-reported health' is not informative. Do the researchers want to say 'self-reported health impacts?' Please revise it. In addition, it is also attractive to say, 'engagement in healthy behaviors' instead of 'self-care behaviors.'

Thanks for the opportunity to clarify. We have modified these terms to read as "self-reported health outcomes" and "engagement in healthy behaviors" throughout the manuscript.

14) It is not good to use abbreviations or acronyms in the title. Hence, instead of the Acronym 'U.S.' write in full words 'United States.'

Thank you for this comment. We will defer to editorial discretion on this point; a cursory search of recent PLOS One articles has shown this abbreviation is common in manuscript titles.

15) Please revise the abstract importance and objectives [to address #13]

Done.

16) In the abstract, it would be good to include the summary of analyses that researchers carried out.

Thank you for this comment. We are unfortunately constrained regarding word count for the abstract (300 words) and cannot provide a full description of our analyses but have attempted to provide some additional detail. Our revised abstract now reads:

"Design: Interrupted time series stratified by age, gender, race/ethnicity, educational attainment, household income, and employment status.

Setting: United States.

Participants: All adult respondents to the 2016-2020 Behavioral Risk Factor Surveillance System (N=2,146,384).

Exposure: Survey completion following the U.S. public health emergency declaration (March-December 2020). January 2019 to February 2020 served as our reference period.

Main Outcomes and Measures: Self-reported health outcomes included the number of days respondents spent in poor mental health, physical health, or when poor health prevented their usual activities of daily living. Self-reported engagement in healthy behaviors included the mean number of hours slept per day and participation in any exercise."

17) The first sentence of the Introduction needs revision to: "The novel coronavirus (COVID-19) began to spread across the world in December 2019 and on March 13, 2020. In response to the pandemic, different countries of the world had been taking different prevention and control measures. Similarly, the United States declared a national public health emergency."

Thank you for this comment, we have revised the first few sentences of our Introduction for clarity:

"The novel coronavirus (COVID-19) began to spread across the world in December 2019 and on March 13, 2020, the United States declared a national public health emergency. Efforts to mitigate the spread of the virus included social distancing, stay-at-home orders, closure of nonessential business, travel restrictions, and quarantines. Additionally, the Centers for Disease Control (CDC) and Centers for Medicare and Medicaid Services advised hospitals across the United States to prioritize urgent visits and delay elective or noncritical medical services to diminish the spread of COVID-19 in health care settings."

18) *The last paragraph of the Introduction needs revision. Indeed, the researchers should explain the gaps in detail and convince why this study is needed and its input to the existing literature.*

Thank you, we have revised the Introduction to better explain the gaps and how we address them:

"Prior work on pandemic's health impacts generally focused on the acute or long-run health effects of COVID-19 infection. To our knowledge, there have been no prior work documenting population-level health effects during the pandemic. Certain features of the pandemic may mitigate against negative health impacts due to deferred/avoided care. Prior work suggests that individuals might adopt healthier lifestyles during periods of unemployment or reduced economic activity. These may include, but are not limited to, increased exercise participation or mean hours of sleep per day. Additionally, the pandemic triggered a recession that may lead to concomitant reductions in job-related stress and hazardous working conditions for some adults. Thus, the net effect of the pandemic on population-level physical and mental health is unclear."

19) *Our objectives were........no need of making separate paragraphs for the objective. In addition, no need of listing them. Rather write it in a single sentence and merge it with the last paragraph of the introduction section.*

Done.

20) *Some variables need Operational definitions and explain how they were measured. Please define poor mental health, poor physical health, and exercise. Explain in detail how these variables were measured.*

Thank you for this comment. The complete list of our outcomes included exact question texts and response formats are provided in Table 1 of the revised manuscript.

21) *Did the researchers check assumptions for the statistical tests carried out? What are the findings of assumptions? Any violation?*

Our analyses included t-tests and ordinary least squares regression models, both of which are robust to even serious deviations from normality. However, we note in the methods that we used heteroskedasticity-robust standard errors. The BRFSS is a repeated cross-sectional survey and not a panel, however we also clustered our standard errors by state to account for pandemic-related policies that may affect their residents. We note this in our revised Methods:

"We employed heteroskedasticity-robust standard errors in all regression models. Errors were also clustered by state to account for pandemic-related policies that may affect their residents."

22) *The researchers showed the trends in poor physical and mental health days (figure 1). That is good. Would you please explain the trends of self-care behaviors/engagement in health behaviors during the COVID-19 pandemic?*

Absolutely! These figures are now available in Appendices A2 & A3.

23) *The discussion is shallow. The researchers should discuss the pertinent findings from both theoretical and empirical perspectives.*

Thank you for this comment; we have substantially expanded our Discussion. For details of these changes, please see our responses to specific points outlined in #8, #9, #10.
