# Supplementary material for: Trends in U.S. self-reported health and self-care behaviors during the COVID-19 pandemic
Source: PLoS One. 2023 Sep 19;18(9):e0291667. doi: 10.1371/journal.pone.0291667 (PMC10508610; doi:10.1371/journal.pone.0291667)
Supplement: Attachment — Submitted filename: PLOS Response Letter 2023 08 31.pdf [file pone.0291667.s004.pdf]

VANDERBILT UNIVERSITY School of Medicine

Department of Health Policy

August 31st, 2023

Dear Dr. Kebede,

Thank you for the opportunity to revise and resubmit our manuscript, "Trends in U.S. self-reported health and self-care behaviors during the COVID-19 pandemic" for consideration at *PLOS ONE*. The reviewers' and editor's comments have been very helpful and strengthened the paper. Our responses are below.

Sincerely,

Kevin N. Griffith, Ph.D.
Department of Health Policy
Vanderbilt University School of Medicine

Reviewer #2 comments:

Some variables such as poor mental health, poor physical health and exercise need to be defined (operational definition) and explain in detail how they were measured.

Thanks for the opportunity to clarify. All outcomes were defined in Appendix A1 including the exact survey text and response format; we have moved this to the main text (Table 1) to aid the reader.

Please describe the assumptions that the researchers checked for the statistical tests carried out. Please incorporate the findings of assumptions in the method section.

Thank you for this question. We estimated ordinary least squares regressions (OLS) for all analyses. We estimated a total of 182 regression models (26 population groups x 7 outcomes) and it would not be possible to present all of the diagnostic regression output. There are 5 key assumptions for OLS:

1) Linearity of regressors: All of our independent variables are binary, thus this assumption does not need to be checked.
2) Normality of model residuals. As stated in the Methods section: "Due to the large sample size and our interest in population average effects, we opted to conduct linear probability models using ordinary least squares to aid in interpretability of results." Further, we include the following citation which describes how the normality assumption is not important for analyses of large public health datasets:

Lumley T, Diehr P, Emerson S, Chen L. The importance of the normality assumption in large public health data sets. Annu Rev Public Health. 2002;23. doi:10.1146/annurev.publhealth.23.100901.140546

3) Homoscedasticity of model residuals. We also state in the Methods section: "We employed heteroskedasticity-robust standard errors in all regression models." This obviates the need to check this assumption.
4) Independence of observations. The BRFSS is a repeated cross-sectional survey with a new sample of respondents each year. The primary risk here is that residents in the same state are subjected to the same set of laws and policies (e.g., shelter in place orders). Thus, our Methods section includes the following which addresses this concern: "Errors were also clustered by state to account for pandemic-related policies that may affect their residents."
5) No multicollinearity. Multicollinearity is usually obvious to diagnose because the standard errors of your regression explode if it is present. However, we also checked the variance inflation factor (VIF) for each regression specification. The VIF did not exceed 10 in any of our models, thus multicollinearity was not an issue.

We defer to the editor for whether this information should be included in the manuscript text versus the peer review tab, since the former is unusual.
