# Supplementary material for: Trends in U.S. self-reported health and self-care behaviors during the COVID-19 pandemic
Source: PLoS One. 2023 Sep 19;18(9):e0291667. doi: 10.1371/journal.pone.0291667 (PMC10508610; doi:10.1371/journal.pone.0291667)
Supplement: Attachment — Submitted filename: Reviewers comments, June, 2023.docx [file pone.0291667.s002.docx]

**Trends in U.S. self-reported health and self-care behaviors during the COVID-19
pandemic**

This is a valuable article and can add input to the existing literature. However, there are some points that need clarity. Please find the comments below:

**Comments**

**Title**:

1. The term ‘self-reported health’ is not informative. Do the researchers want to say ‘self-reported health impacts’? Please revise it. In addition, it is also attractive to say ‘engagement in healthy behaviors’ instead of ‘self-care behaviors’.

2. It is not good to use abbreviations or acronyms in the title. Hence, instead of the Acronym ‘U.S.’ write in full words ‘United States’.

**Abstract**:

Importance: However, it remains unclear how the COVID-19 pandemic impacted self-reported mental and physical health and influence human behaviors.

Objective: please revise it. To assess changes in self-reported health outcomes/impacts during the COVID-19 pandemic and trends of engagement in health behaviors.

In abstract section; good to include the summary of analyses that researchers carried out.

**Introduction**:

1. The first sentence needs revision. The novel coronavirus (COVID-19) began to spread across the world in December 2019 and on March 13, 2020. In response to the pandemic, different countries of the world had been taking different prevention and control measures. Similarly, the United States declared a national public health emergency.

2. The last paragraph of the introduction needs revision. Indeed, the researchers should explain the gaps in detail and convince why this study is needed and its input to the existing literature.

3. Our objectives were........no need of making separate paragraphs for the objective. In addition, no need of listing them. Rather write it in a single sentence and merge it with the last paragraph of the introduction section.

**Methods**:

1. Some variables need Operational definitions and explain how they were measured. Please define poor mental health, poor physical health, and exercise. Explain in detail how these variables were measured.

2. Did the researchers check assumptions for the statistical tests carried out? What are the findings of assumptions? Any violation?

**Results**

**1**. The researchers showed the trends in poor physical and mental health days (figure 1). That is good. Would you please explain the trends of self-care behaviors/engagement in health behaviors during the COVID-19 pandemic?

**Discussion**

The discussion is shallow. The researchers should discuss the pertinent findings from both theoretical and empirical perspectives.
